# Comparative Analysis of the Functional Properties of Films Based on Carrageenans, Chitosan, and Their Polyelectrolyte Complexes

**DOI:** 10.3390/md19120704

**Published:** 2021-12-12

**Authors:** Aleksandra V. Volod’ko, Viktoriya N. Davydova, Valentina A. Petrova, Dmitry P. Romanov, Evgeniya A. Pimenova, Irina M. Yermak

**Affiliations:** 1 G.B. Elyakov Pacific Institute of Bioorganic Chemistry, Far Eastern Branch, Russian Academy of Sciences, Prospect 100 Let Vladivostoku 159, 690022 Vladivostok, Russia; vikdavidova@yandex.ru (V.N.D.); imyer@mail.ru (I.M.Y.); 2 Institute of Macromolecular Compounds, Russian Academy of Sciences, Bolshoi Pr. VO 31, 199004 Saint Petersburg, Russia; valentina_petrova_49@mail.ru; 3 I.V. Grebenshchikov Institute of Silicate Chemistry, Russian Academy of Sciences, Adm. Makarova Emb., 2, 199034 Saint Petersburg, Russia; dprom@mail.ru; 4 A.V. Zhirmunsky National Scientific Center of Marine Biology, Far Eastern Branch, Russian Academy of Sciences, Palchevskogo 17, 690041 Vladivostok, Russia; eapimenova@yandex.ru

**Keywords:** carrageenan, chitosan, film, polyelectrolyte complex, mucoadhesive polymer

## Abstract

The influence of the structural features of carrageenan on the functional properties of the films was studied. The carrageenans and chitosan films, as well as three-layer films containing a polyelectrolyte complex (PEC) of the two, were prepared. The X-ray diffractograms of carrageenan films reflected its amorphous structure, whereas chitosan and three-layer films were characterized by strong reflection in the regions of 20° and 15° angles, respectively. The SEM of the cross-sectional morphology showed dense packing of the chitosan film, as well as the layer-by-layer structure of different densities for the PEC. Among the tested samples, κ/β-carrageenan and chitosan films showed the highest tensile strength and maximum elongation. Films containing the drug substance echinochrome were obtained. Mucoadhesive properties were assessed as the ability of the films to swell on the mucous tissue and their erosion after contact with the mucosa. All studied films exhibited mucoadhesive properties. All studied films exhibited mucoadhesive properties which depended on the carrageenans structure. Multilayer films are stronger than single-layer carrageenan films due to PEC formation. The resulting puncture strength of the obtained films was comparable to that of commercial samples described in the literature.

## 1. Introduction

Thin films are a novel drug delivery tool and have been used as an alternative dosage form [1]. They may be useful for reducing extensive metabolism and eliminating the side effects of a drug [2]. Thin films have shown the capabilities of enhancing drug efficacy and reducing the dosing frequency [3], all of which make them versatile platforms for drug delivery. Ideal thin films should be nontoxic, biocompatible, and biodegradable. They must have a sufficient drug-loading capacity and acceptable formulation stability, a fast dissolution rate, or a long residence time at the site of administration [4]. This delivery system has been used for both systemically and locally via several pathways, including the oral, buccal, sublingual, ocular, and transdermal routes of administration.

The marine hydrobionts are a renewable source of a varied polysaccharides, which can easily be utilized to develop various drug delivery systems (DDS), owing to their biocompatible, biodegradable, and nontoxic nature. Localized and prolonged effects of active ingredients included in DDS can be provided due to the mucoadhesive properties of matrices. Polysaccharides that hold promise as delivery systems are sulphated polysaccharides of red algae—carrageenans (CRGs), which have good mucoadhesive properties and the ability to form gels or increase the viscosity of solutions [5] and form complexes with polycations [6].

CRG is a sulphated galactose copolymer composed of alternating 3-linked β-D-galactopyranose (G-units) and 4-linked α-D galactopyranose (D-units) or 4-linked 3,6-anhydro-α-D-galactopyranose (DA-units), forming the disaccharide repeating unit of CRGs [7]. CRG is classified into various types, depending on the presence or absence of 3,6-anhydro-galactose units and the number and location of sulphate groups [8]. CRGs are approved for use in the food and medical industry and have already been included in the US Pharmacopeia 35- National Formulary 30 S1, British Pharmacopeia 2012, and European Pharmacopeia 7.0; thus, they have a promising future in the pharmaceutical industry [9].

Kappa-, iota-, and λ- are the most important types of CRGs applied in the pharmaceutical and commercial fields. Kappa- and iota- CRGs form a three-dimensional double-helix network via crosslinking of adjacent sulphate groups. The sulphate groups of λ-CRG do not undergo crosslinking and thus do not form gels [10]. Most algae contain hybrid CRGs, the polymer chain of which can be built from disaccharide units of various types. [9,11].

CRGs are used as a matrix for DDS. An intravaginal film platform (consisting of polyvinyl alcohol and λ-CRG) for targeted delivery of small interfering RNA-loaded nanoparticles to dendritic cells as potential gene therapy for the prevention of human immunodeficiency virus infection was reported [12]. The combination of λ-CRG and gelatine in different ratios is useful in modulating the drug release profiles and the mucoadhesive and rheological properties of hydrated formulations [13]. Sipahigil et al. prepared kappa-CRG beads as a controlled release system for the slightly water-soluble drug ibuprofen and a freely water-soluble drug verapamil hydrochloride [14]. As shown by us earlier, CRG increases the solubility of echinochrome A (ECH; the substance of the drug Histochrome^®^) and forms complexes with it, which are highly gastroprotective [15].

Chitosan (CH) was one of the first carbohydrate polysaccharides with mucoadhesive properties to be proposed as a vehicle. CH can trigger a reversible opening of tight junctions between epithelial cells and thereby facilitate the paracellular transport of large, hydrophilic compounds [16]. CH, a cationic (1–4)-2-amino-2-deoxy-β-d-glucan with a degree of acetylation lower than 0.40, is predominantly produced from marine chitin [17]. The use of CH as a carrier containing encapsulated, dispersed, adsorbed, or conjugated medicinal products makes it possible to reduce the dosages of active substances, increase their therapeutic effect, and increase the safety of their application [18]. CH is widely used to produce polyelectrolyte complexes (PECs), including to obtain multilayer composites with polysaccharide polyacids [19,20]. CH-based PECs can be used as a drug delivery system because PEC formation can control mechanical strength, swelling behavior, and delayed digestibility to ensure the effective and sustained release of encapsulated drugs [21].

Comfortable and flexible films containing a polymeric blend would be a promising platform for drug delivery in the oral cavity [22]. There has been a rise in the development of buccal films as alternative drug delivery for various classes such as analgesics, anti-inflammatory, and anesthetic drugs, proteins and peptides. Mucoadhesive films have been used as a platform for transmucosal buccal delivery of drugs targeting opioid analgesics such as fentanyl citrate (Onsolis^®^/Breakyl^®^) for treating immense pain [1]. Similarly, the mucoadhesive film remains attached to the buccal area without showing any erratic absorption profile, resulting in less inter- and intra-individual variability [23].

The films containing PEC of CH and CRG are presented in the literature mainly for commercial types of κ- and ι-CRGs. These films are mainly used as a prospect of application in the food packaging [24] or to modify surface [25,26]. Films obtained on the basis of individual polysaccharides do not always have the required functional properties. Often, to improve the characteristics of the films, various additives [27] are added. This can change the biological properties of the obtained materials. The mechanical/mucoadhesive characteristics of films, and especially their relationship with the structure of the polymers used, have not been sufficiently studied. The use of various types of CRGs with an established chemical structure makes it possible to study the effect of the structural features on the characteristics of the resulting films. This is important for the development of specific systems with the desired properties, particularly of various DDS based on them.

The method of mixing initial polysaccharide solutions with further evaporation of the solution from the mixture [28] or sequential immersion of a solid support in polysaccharide solutions [29,30,31] used often to obtain of PEC films. The substrate-independent multilayer films, obtained in this work, have different surface properties. The study of their surface, as well as the intermediate layer of PEC, makes it possible to expand the areas of their application. This work aimed to study the influence of the structural features of CRG the functional properties (mechanical strength, crystallinity, and ability to swell on mucous tissue) of films obtained based on three types of CRGs with different monosaccharide compositions (the presence/absence of 3,6-anhydro-α-D-galactopyranosyl residues) and different degrees of sulfation, as well as their complexes with CH. To this end, the following parameters of the films were determined: strength and energy per puncture, as well as elongation. Using the mucous tissue of the intestine of a pig, the ability of the films to swell on the mucous tissue and their adhesion/erosion after contact with the mucosa were evaluated. To assess the feasibility of using the obtained films for mucosal drug delivery, matrices containing ECH [32] were produced. The creation of new forms of delivery systems ECH as films may be important for the noninvasive use of this drug. The functional properties were also evaluated after the inclusion of ECH in the polysaccharide matrices.

## 2. Results and Discussion

### 2.1. Characteristics of the Polysaccharides

The CRGs were extracted from red seaweeds *C. armatus* and *T. crinitus*, purified from low-molecular-weight impurities and precipitated from solutions by alcohol as described earlier [33]. Crude polysaccharides were fractionated with KCl into the gelling (KCl-insoluble) and the nongelling (KCl-soluble) fractions. The structures of the polysaccharide fractions were studied by NMR and FTIR-spectroscopy. Absorption bands of FTIR spectra and chemical shifts of NMR spectra-obtained polysaccharides were compared with signals of known CRG structures [8,34] and with the spectra of polysaccharides isolated from *C. armatus* and *T. crinitus* by us earlier [33,35].

**Figure A1:**
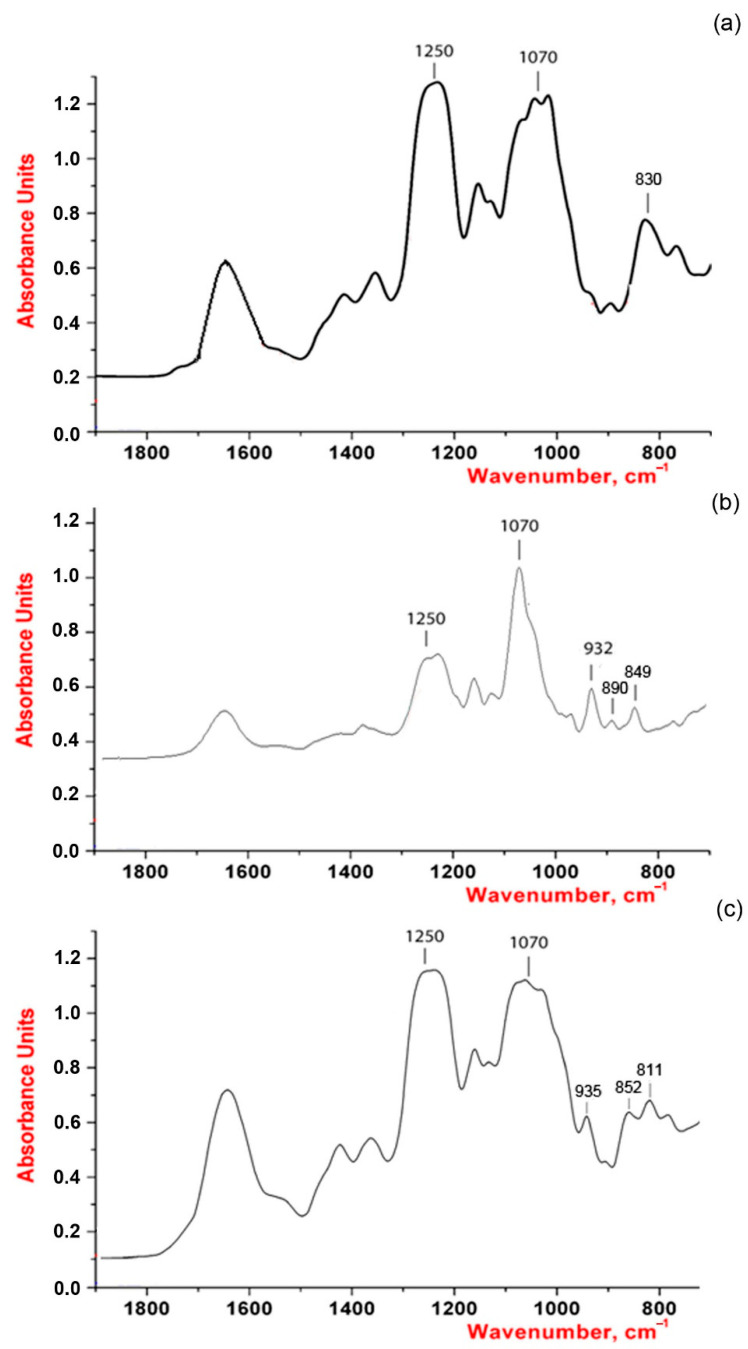
IR spectra (**a**) λ-CRG, (**b**) κ/β-CRG, and (**c**) x-CRG.

**Figure A2:**
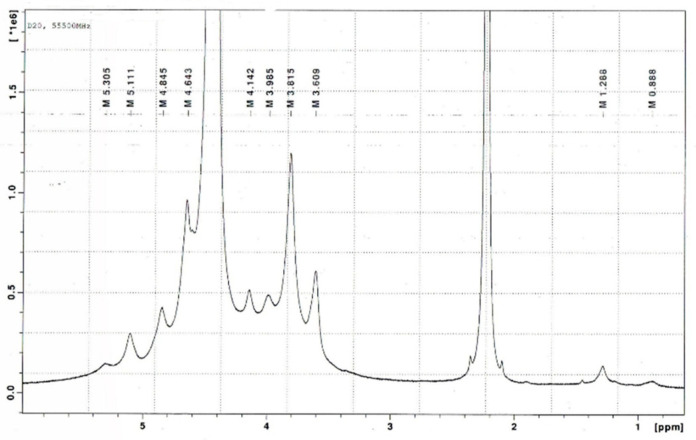
The ^1^H-NMR spectrum of gelling polysaccharide from *T. crinitus*.

IR spectra of the polysaccharides (Figure A1), the ^1^H- and ^13^C- NMR (Table A1, Figure A2), spectra of the KCl-insoluble fraction of *T. crinitus* polysaccharide were presented.

In the ^13^C NMR spectrum of gelling polysaccharide from *T. crinitrus*, there were poorly resolved signals at 103.1 and 102.7 ppm, which is the result of overlapping of the C-1 signals of the 3-linked β-D-galactose 4-sulfate of the κ-CRG (G4S) and the 3-linked β-D-galactose (G) of β-CRG. The signal at 95.1 ppm and less intense signal at 94.4 ppm, among the six signals observed in the anomeric carbon resonance region of the ^13^C NMR spectrum of *T. crinitus* polysaccharide were characteristic of C-1 of the 4-linked 3,6-anhydro-α-D-galactose (DA) of κ- and β-CRG (Table A1, Figure A2).

An absorption band in the region of 1250 cm^−1^ in the IR spectra of all studied CRGs (Figure A1) indicated the presence of sulfate groups (–S=O asymmetric vibration). However, in the IR spectra of gelling polysaccharides *T. crinitus* (Figure A1b), this band was not intense, which indicates an insignificant content of sulfate groups in this sample. Absorption bands at 932 and 849 cm^−1^ in IR spectra of polysaccharide were characteristics of 3,6-anhydrogalactose (C–O vibration) and the secondary axial sulfate group at C-4 of the 3-linked β-D-galactose residue, respectively. This made it possible to assign the polysaccharides to κ-CRG. Moreover, the absorption band at 890 cm^−1^ in the IR spectrum of the gelling polysaccharide of *T. crinitus* also evidenced the presence of nonsulfated β-D-galactose residues, typical for β-CRG. FTIR spectroscopy data suggest that gelling polysaccharide from *T. crinitus* had a hybrid κ/β-structure [36]. According to data obtained by FTIR and NMR spectroscopy, gelling polysaccharides from *T. crinitus* had hybrid κ/β structures.

In the middle range of the IR spectra of nongelling polysaccharides *T. crinitus* (Figure A1c), one can observe a very intense absorption band at about 1250 cm^−1^ characteristic of the sulfated ester group. This band is associated with lower-frequency absorption bands at 852 and 811 cm^−1^ I, suggesting that sulfated ester group may have a different position along the polysaccharide chain. These can be in particular C-4 and C-2 of the 3-linked β-D-galactose residues. A characteristic band attributed to the 3,6-anhydro bridge vibration at 935 cm^−1^ was also clearly identified in the spectrum. Thus, its FTIR spectrum was identical to the IR spectrum of a nongelatinous polysaccharide from sterile alga *T. crinitus*, the structure of which is based on a repeating disaccharide unit consisting of β-(1→3)-D-galactopyranoside-2, 4-disulfate, and α-(1→4)-3,6-anhydro-D-galactopyranoside [36].

There was no absorption band corresponding to 3,6-anhydrogalactose in the IR spectrum of soluble fraction of *C. armatus* (Figure A1a). By contrast, there was a wide absorption band at 820–830 cm^−1^ corresponding to the primary equatorial sulfate group at C-6 and the secondary equatorial sulfate group at C-2 of 4-linked α-D-galactose, which were characteristic of λ-CRG [33]. It appears that a small amount of the biosynthetic precursor of κ-carrageenan is also present in the soluble fraction. Thus, the FTIR spectroscopy data indicated that KCl-soluble fraction from *C. armatus* was represented mainly by λ- (G2S-D2S,6S—carrabiose) with other carrageenan types.

**Figure A3:**
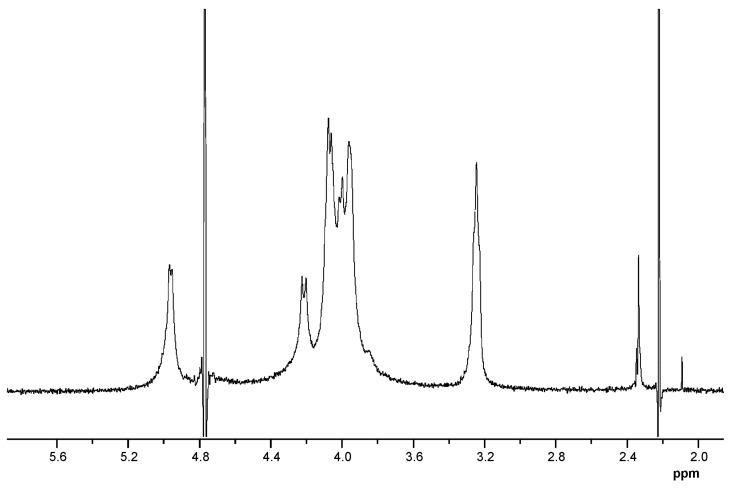
The ^1^H-NMR CH spectrum.

The structures of the disaccharide repeating units of the CRGs and molecular weights of CRGs and elemental composition are shown in Table 1. Thus, CRGs used for film preparation differed by the presence (κ/β and x-CRGs) or absence (λ-CRG) of 3,6-anhydro-D-galactose, as well as the location and number of sulphate groups. The sulfation degree of CRG decreased in the order λ > x > κ/β. High-molecular-weight CH (MW ~ 480 kDa) was obtained by alkaline deacetylation of chitin from king crab shell. The degree of N-acetylation was calculated from the ratio of the integral intensities of the signals of the three protons of CH3CON-group and the proton at the C-2 atom of the monosaccharide unit. According to the obtained data, the degree of N-acetylation of CH was 7% (Figure A3).

### 2.2. Film Characteristics

Films were obtained by casting a polysaccharide solution onto a balanced flat substrate. The thickness of the films obtained varied within 10–26 microns, and the moisture content did not exceed 10%. In this work, both single-layer films obtained based on CRGs and CH and three-layer CRG:PEC:CH were studied. The formation of PEC is a prospective approach to improve the mechanical properties of initial polysaccharides and also to impart other beneficial features to the composite material [37]. The advantage of PEC is that they can be formed without the use of chemical crosslinking agents, thereby making the synthesis process very simple. In our case, CH acts as a polycation due to the presence of free -NH2 group in the D-glucosamine units, and CRG acts as a polyanion. Multilayer CRG:PEC:CH films were obtained from κ/β-, x-, λ-CRGs, and CH using the layer-by-layer deposition method [20]. To confirm the fact of the PEC formation, the films were washed from unbound polysaccharides (according to the Section 3.3.1). The insoluble precipitate of PEC was dried and weighed, and the yield of the complex was calculated. The elemental composition of the insoluble PEC layer is presented (Table 2). The presence of atoms of S and N in this layer, as well as the previously obtained FTIR data [6] confirmed the presence of PEC. According to the obtained results, the highest PEC yield was observed for κ/β-CRG:CH (Table 2).

### 2.3. Mechanical Properties of the Films

**Figure 1:**
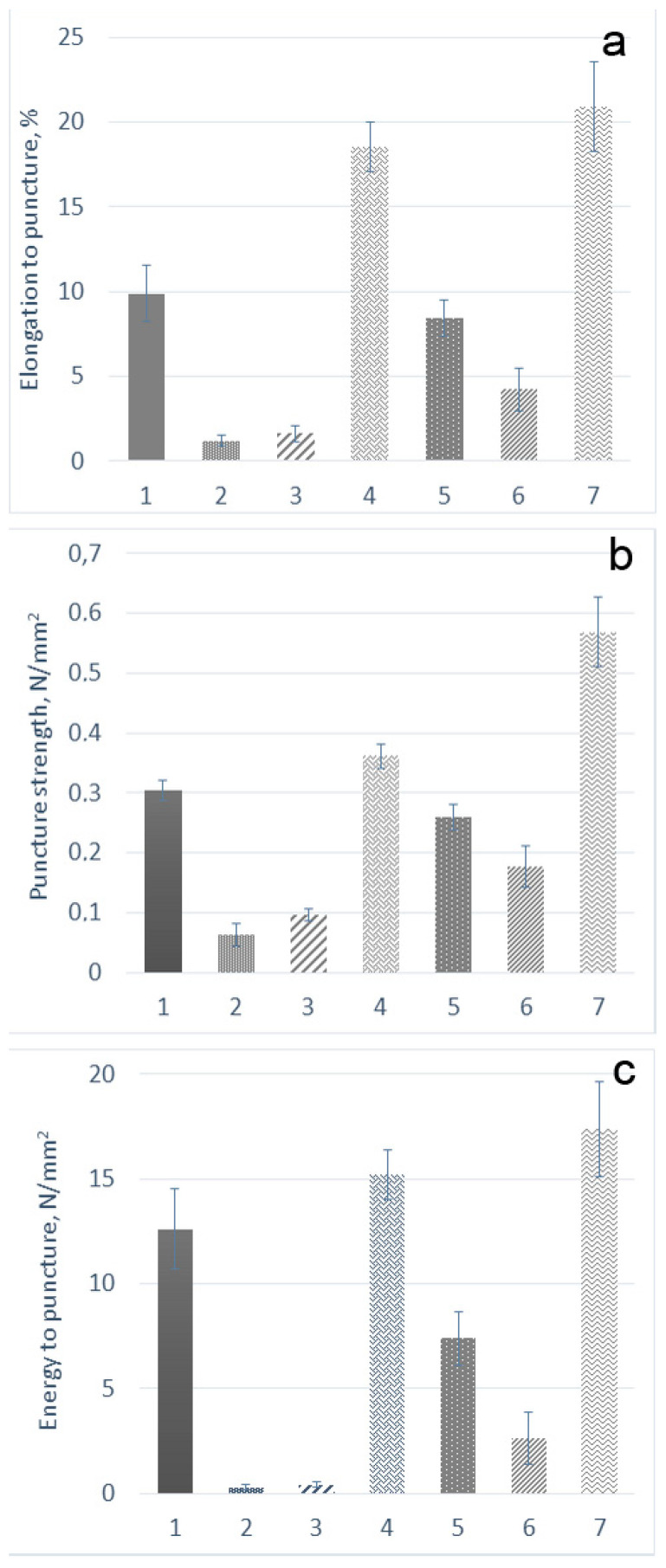
Mechanical properties of the films (**a**) elongation to puncture, (**b**) puncture strength, (**c**) energy to puncture: (1) CH; (2) x-CRG; (3) λ-CRG; (4) κ/β-CRG; (5) x-CRG:PEC:CH; (6) λ-CRG:PEC:CH; and (7) κ/β-CRG:PEC:CH.

At present, a texture analyzer is widely used to characterize mechanical properties. The main characteristic indicators of films during their use are the strength, energy, and elongation to puncture. These parameters were determined by us for the obtained films (Figure 1).

The elongation to puncture, calculated from the penetration depth of the probe, varied from 1% to 20%, which indicates the elasticity of the samples. It is known that soft and weak polymers exhibit low tensile strength and low elongation at break, whereas the hard and tough polymers have high tensile strength and high elongation at break [38]. Among the polysaccharides used in this work, κ/β-CRG formed films with the highest tensile strength. This polysaccharide contains many 3,6 anhydrogalactose and few sulphates. In the κ/β-CRGs, the 4-linked galactose residues, respectively, DA adopted the 1C4-chair conformation. This conformation, which results from the 3,6-anhydro bridge, is crucial for the formation of the helical structure [39,40]. Thus, κ/β-CRG can form a strong and rigid gel. According to Figure 1a, there is a tendency for three-layer films to stretch better than parent CRG films, regardless of type. Among all studied films, the three-layer κ/β-CRG:PEC:CH exhibited the maximum elongation. An increase in the strength and elongation of films in the case of PEC can be associated with an increase in their thickness and probably a change in morphology.

The composition of the films can have a significant effect on their mechanical strength and flexibility, as well as their morphology. The parameter allowing the consideration of changes in the composition of the film is the energy of the puncture. It was calculated to ensure the comparability of the obtained results (Figure 1). Λ- and x-CRG films exhibited the lowest puncture energy and were also less tensile and elongated. Most of the energy was required to puncture the original κ/β-CRG and CH sample. Three-layer films were stronger than films of the original CRG (for x-CRG: CH 4.1 times (26.6 energy per puncture), for λ-CRG: CH 1.8 times (6.2 energy per puncture), κ/β-CRG: CH 1.6 (1.1 energy per puncture) times), but three-layer films with λ- and x-CRGs were not stronger than the original CH film. As the data presented in Figure 1 show, an increase in the strength of the film was associated with the formation of PEC. The strongest mechanical properties of κ/β-CRG film may be associated with ordered supramolecular structure of the polysaccharide [41]. Its three-layer film still showed the maximum strength and energy to puncture; however, the relative increase in its mechanical properties in comparison with the initial polysaccharide was minimal in comparison with three-layer films of x- and λ-CRGs. Films with a higher degree of pairing between the polycation and the polyanion or covalent crosslinks were shown to have increased rigidity, a reduced film breakdown rate and reduced swelling in aqueous media [42]. According to our previous results [6], CH binds to the maximum amount of sulphate groups in x- and λ-CRGs. As a result, there was a significant increase in the mechanical properties of PEC films, compared with films of the initial polysaccharides, which was probably due to the large number of bonds between CH and sulphate groups of these CRGs. An increase in the thickness of the film did not have a decisive effect on the mechanical characteristics of the sample.

The observed differences in the mechanical properties of films prepared on different types of CRGs were related to differences not only in the primary structure of the polysaccharides but also (and probably to a greater extent) to differences in their macromolecular organization, i.e., their secondary structure. According to previously obtained data, κ/β-CRG at a concentration of more than 25 µg/mL forms a fibrous network structure [41]. X-CRG forms a honeycombed structure at a low concentration (10 µg/mL), but at C = 50 µg/mL, an ordered morphology arises with a large cell-like structure. Λ-CRG differs from this type of polysaccharide by a high degree of sulphation and the absence of 3,6–anhydrogalactose. The conformation of both the galactose residues of a disaccharide unit corresponds to the 4C1-chair conformation, and ordered helices are not formed by this structure [39,40].

Preis et al. [43] used the puncture test to evaluate marketed and newly developed film products. According to their data, the elongation to break of the marketed films was 1.03%–6.54%, and that of the prepared film samples from different derivatives of cellulose was 4.51%–33.17%. According to the author, a puncture strength of at least 0.08 N/mm^2^ confirmed damage-free handling and also industrial production of films that could be used for pharmaceutical specifications. Thus, the mechanical properties of the obtained films were similar to those of commercial samples of films and reference soft tissues. The resulting puncture strength of the films was higher than 0.08 N/mm^2^, which indicates the possibility of using our films for buccal delivery.

### 2.4. X-ray Diffraction Study

**Figure 2:**
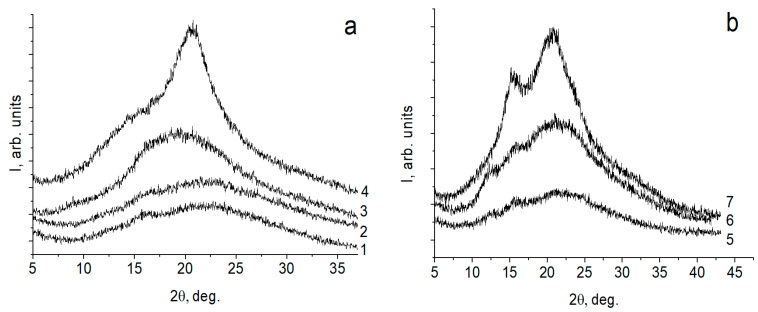
X-ray diffractograms of the samples obtained in the reflection mode (**a**) one-layer films: (1) λ-CRG; (2) x-CRG; (3) κ/β-CRG; (4) CH, (**b**) three-layer CAR:PEC:CH films: (5) λ-CRG:PEC:CH; (6) x-CRG:PEC:CH; and (7) κ/β-CRG:PEC:CH.

The diffractograms of the initial polymer films, the three-layer CAR:PEC:CH films, are presented in Figure 2. The diffractograms of CRGs reflect the amorphous structure of these polymers. The X-ray diffraction pattern of a CH film obtained from a solution in acetic acid is characterized by a strong reflection in the region of the angle 2θ = 20°. For three-layer films, the appearance of reflections in the diffractograms in the region of the angle 2θ = 15° (the most intense for the composite with κ/β-CRG) was observed. As shown in previously published works, during the formation of polylayer composites based on CH and polysaccharide polyacids, the formation of PEC between the polymer layers initiates the transformation of the hydrated polymorphic CH modification into an anhydrous one [20,44,45]. This is accompanied by the appearance of a reflection in the diffractograms in the region of the angle 2θ = 15°. The degree of CH structuring depends on the degree of realization of intermolecular contacts. Our data indicate that the PEC layer forms a sparser physical network in the λ- and x-CAR with CH multilayer films than in the κ/β-CAR:CH pair.

### 2.5. Mucoadhesive Properties of Films

Mucoadhesive polymers (MAPs) are hydrophilic networks that contain multiple polar functional groups. Such polymers interact with mucus not only through physical entanglements but also through secondary chemical bonds, resulting in weakly crosslinked networks.

**Figure 3:**
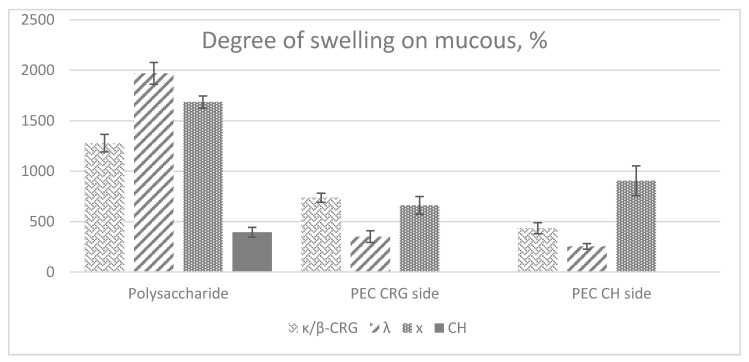
Degree of swelling on mucous one-layer polysaccharide films and three-layer CRG:PEC:CH films from CRG side or CH side.

The swelling of the polymers is known to be the fundamental step required for bioadhesion [46]. The degree and rate of swelling play a key role in controlling drug release. These parameters can be considered as the indicator for mucoadhesive potential and drug release profiles [47]. The swelling properties of the films, that is, their ability to absorb water, are measured by assessing the percent hydration. The measurement of the degree of hydration plays an important role in providing information on mucoadhesive strength of the polymeric film. The degree of swelling on mucosal surface single-layer polysaccharide films: CH, κ/β-, λ- and x-CRGs, as well as three-layer films: κ/β-CRG:PEC:CH, λ-CRG:PEC:CH and x-CRG:PEC:CH using the inner surface of the small intestine of a pig was studied (Figure 3). Films were applied on the mucosal surface on both sides (by the CH and the CRG sides).

Single-layer CRG films had a higher degree of swelling than CH films (Figure 3). The low degree of swelling of CH is probably due to the degree of crystallization of its structure, which is evidenced from the X-ray (Figure 2, line 4) and its ability to form strong intermolecular associations [48,49]. The swelling value correlated with the number of sulphates in polysaccharides and decreased in the λ > x > κ/β- CRG series. This can also be explained by the difference in their macromolecular organization. Κ/β- and x-CRGs contain 3,6-anhydrogalactose, which contributes to the formation of a three-dimensional network structure, the packing density of which is lower in the case of the x-CRG. This structure prevents the penetration of the solvent into it. The random coil structure of λ-CRG, bearing a large number of hydroxyl and sulphate groups on its surface, ensures its swelling on the surface of the mucin layer. This can be the result of its disordered secondary structure (random coil, which promotes a higher degree of hydration of the polysaccharide).

The hydration of polymers is the reason for relaxation and interpenetration of the polymeric chain. However, overhydration results in a decrease in mucoadhesion properties due to the formation of slippery mucilage [50]. In our case with λ-CRG, the film swelled and lost its shape.

It is interesting to note that three-layer films do not show such high values of hydration as the CRG films; moreover, the three-layer λ-CRG:PEC:CH film loses its ability to swell on the mucous membrane by 5.6 times, while x-CRG:PEC:CH and κ/β-CRG:PEC:CH only lose such an ability by 2.5 and 1.7 times. During the formation of PEC, the number of free charges in polysaccharides decreases, which causes a decrease in film swelling. The λ-CRG: PEC:CH film on the CH side slightly loses its ability to swell on the mucous membrane (1.6 times less than a single-layer CH film). In the case of x-CRG:PEC: CH and κ/β-CRG: PEC:CH films this ability increases (2.3 and 1.1 times more than a single-layer CH film, respectively). This behavior is well correlated with the mechanical properties of the films (Figure 1). Thus, films of x- and λ CRGs, which have the lowest puncture strength and energy, show the greatest swelling. Similarly, an increase in the mechanical properties of films due to the formation of PEC leads to a decrease in their swelling ability.

At the second stage of MAP adhesion, contacts are established between the biological tissue and biopolymer as a result of the diffusion of sufficiently flexible polymer chains into gaps, loops, and pores of the glycoprotein network (diffusion theory) or as the formation of bonds between it and the mucin glycoprotein (adsorption theory) [51]. Thus, the gradual or partial destruction of the film could be expected, which was observed in the case of a single-layer λ-CRG film, as well as a three-layer λ-CRG:PEC:CH film on the CRG side (Table 3). However, the weight of the films for CH, x- and κ/β-CRG, as well as their three-layer films with CH after contact with the mucosa, had increased. This can occur when part of the mucin layer is removed along with the film, probably due to the strong interaction of mucus components with polysaccharides. Schoeler et al. [52] inspected the morphology and internal structure of the films, revealing that the helical conformation adopted by CRG in solution is kept during the multilayer buildup. Reynaers [53] showed that 3,6-anhydrogalatose-containing iota-CRG has a persistence length of approximately 74 nm. Λ-CRG strongly differs in chain rigidity and persistence length: at 0.1 M NaCl, the persistence lengths of λ-CRG are approximately 13 [54]. Thus, one would expect that although x- and κ/β-CRGs differ in structure, both form less mobile double helices combining in a more closely packed net assembly in solutions due to the presence of 3,6-anhydrogalactose residues. These facts explain the slow diffusion of κ/β- and x-CRGs into the mucus layer and rapid penetration of λ-CRG into the mucin layer due to the mobility of polymer chains. In the case of λ-CRG, binding to CH partially slowed down the destruction of the film. Polysaccharide molecules in PEC, having less mobility, did not leave the polysaccharide matrix and were held more firmly on the mucin layer, which probably did not allow them to diffuse into it. It can also be assumed that due to the formation of such strong bonds between the three-layer films and mucin, part of the mucin layer was removed from the mucous tissue when the film was removed, which increased their mass.

### 2.6. Characterization of the ECH-Loaded Films

The red pigment of water-insoluble ECH from sea urchins is the water-insoluble compound, which is the active substance in the Histochrome^®^ [32]. It has been established that ECH dissolves in aqueous solutions of CRGs. CRGs modify the action of ECH, by decreasing its proinflammatory effect [55].

In this work, ECH was included in the films, and its main characteristics were studied. For this, single-layer films of CH, κ/β-, and x-CRG, as well as three-layer films containing PEC of CH with κ/β- and x-CRG, were chosen. These CRGs contained 3,6 anhydrogalactose, differed in the number and location of sulphate groups (Table 1), and showed the greatest strength properties (Figure 1). Unloaded films were compared with ECH-loaded films.

**Figure 4:**
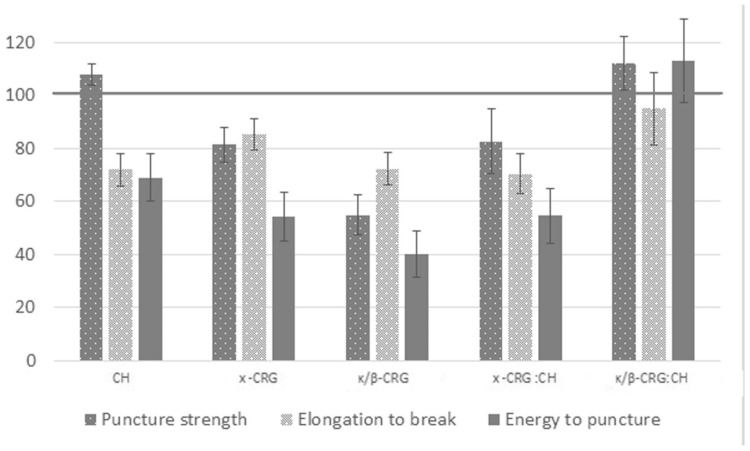
Mechanical properties of ECH-loaded films in comparison with unloaded films, %.

The results of a comparative analysis of the mechanical properties of ECH-loaded and unloaded films are shown in Figure 4. The corresponding indicator of the mechanical properties of an unloaded film was taken as 100%. The introduction of ECH into the polysaccharide film weakens its mechanical properties. As shown by us earlier, ECH interacted with CRG according to an ionic mechanism and probably due to the intermolecular hydrogen bonds formed by a large number of hydroxyl groups present in both CRG and ECH [15]. Most likely, the inclusion of ECH into the polysaccharide matrix and its interaction with CRG loosened the ordered structure of the polysaccharide, which causes a decrease in the film strength. Moreover, this is most typical of κ/β-CRG, which has a more ordered supramolecular organization in comparison with x-CRG [41]. In the case of CH, there is no apparent decrease in film strength; however, a decrease in the value of the puncture energy and elongation to break is observed, taking into account changes in composition and elongation to break. The weakening of the mechanical characteristics of three-layer films was observed only in the case of x-CRG (ECH):PEC:CH, whereas the mechanical properties of a three-layer film with κ/β-CRG containing ECH did not differ statistically significantly from those of the unloaded film.

### 2.7. SEM

The surface properties of films play an important role in drug transport through the mucosa. Light microscopy, scanning electron microscopy (SEM), transmission electron microscopy, and related imaging techniques can be used to observe various surface characteristics such as the surface texture, thickness, and drug distribution (aggregated or scattered) of the film [56]. Among these, researchers have adhered to SEM as a reliable method for examining the surface morphology of the films. However, the analysis of the surface morphology of the obtained films did not reveal any visible differences among the studied samples. Therefore, the morphology of the film cross-sections, which could explain the difference in the mechanical properties, was investigated.

**Figure 5:**
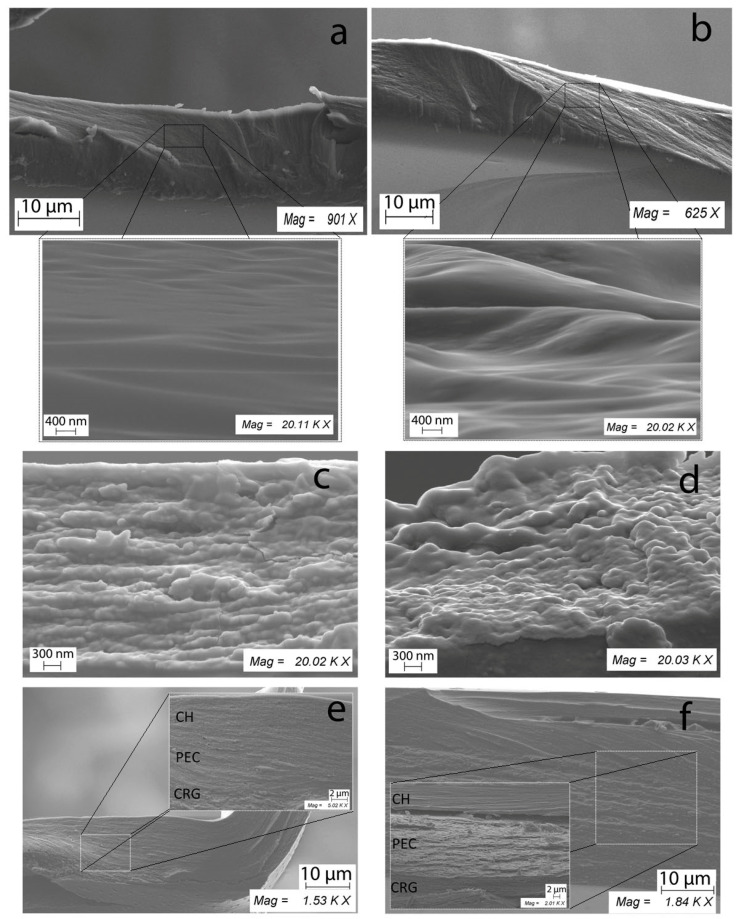
SEM of the cross-sectional morphology of the films: (**a**) CH, (**b**) CH (ECH), (**c**) x-CRG, (**d**) x-CRG (ECH), (**e**) x-CRG:PEC:CH, and (**f**) x-CRG (ECH):PEC:CH.

Figure 5 presents the micrographs of the cross-sectional morphology of the films of CH, x-CRG, and the three-layer film containing their PEC, as well as the same films loaded with ECH at 20,000× magnification by SEM. The micrographs showed that the cross-sectional surface of CH films was homogenous, continuous, compact, and smooth. CRG exhibits uneven and rough surfaces. This explains well the difference in the mechanical properties of the films and the results in the swelling of the films on the mucous tissue. Figure 5a clearly shows the densely packed structure of CH, which justifies its higher energy and puncture strength compared to x-CRG (Figure 1), as well as low swelling of CH film on mucous (Figure 3).

The inclusion of ECH in the CH film leads to the loosening of the structure and more pronounced folds. The film containing ECH showed a more heterogeneous undulating cross-section surface than the CH-based film. In the case of the CRG (ECH) film, scale structures were observed.

Composite films (CRG:PEC:CH) showed multilayer structures. The smooth layer of CH was visible. The PEC and CRG layers were rougher and less densely packed. On scans of a three-layer film with ECH, scales structures were observed (which are also visible for CRG (ECH) films). Three-layer films (Figure 5e,f), in contrast to the films of the initial CRG (Figure 5c,d), according to SEM data, were more densely packed structures, which probably explains their higher mechanical properties and a significant decrease in the degree of swelling on the mucous tissue. The introduction of ECH imparted additional roughness to the film, which may be associated with a disturbance of intermolecular interactions between polysaccharide chains.

### 2.8. Swelling on the Mucous Membrane of ECH-Loaded Films

**Figure 6:**
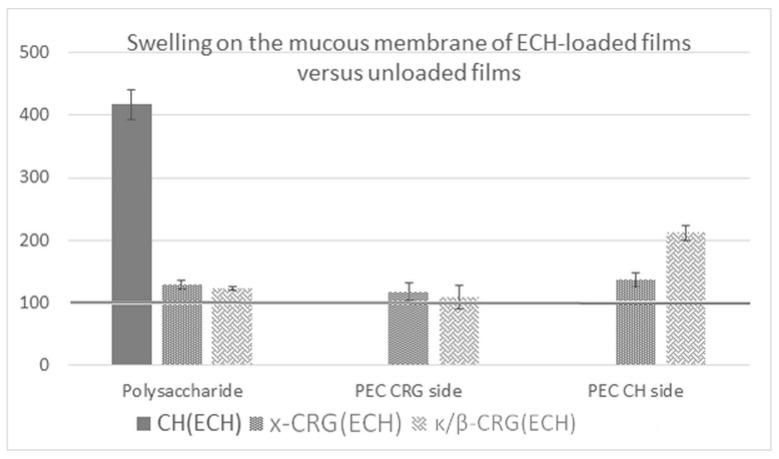
Swelling on the mucous membrane of ECH-loaded films versus unloaded films.

The inclusion of ECH in the films increased their swelling capacity on the mucosal surface (Figure 6). The greatest effect was observed for the CH (ECH) film: the degree of film swelling with ECH increased by a factor of 4.2. In addition, films in contact with the mucous tissue on the CH side showed a high swelling capacity. This may be a result of a change in the supramolecular organization of CH when ECH is included in the film. It can be assumed that in this case, the introduction of ECH can also weaken the intermolecular interactions between the CH chains, loosening the supramolecular structure, which is confirmed by the SEM data (Figure 5b). This in turn facilitates its interaction with mucin due to ionic bonding of the amino groups of CH with negatively charged sialic acid residues present on the mucin.

The relative mass change in films after contact with the mucous membrane for all samples indicates that the films do not disintegrate but are capable of absorbing components of mucous tissue and therefore show good adhesive properties (Table 4).

## 3. Materials and Methods

### 3.1. Polysaccharides

High-molecular-weight CH was obtained by alkaline deacetylation of chitin from king crab shells according to the procedure described earlier [57]. The degree of N-acetylation of the CH sample was calculated according to ^1^H-NMR spectroscopic data according to [58]. It was calculated from the ratio of the integral intensities of the signals of the three protons of CH_3_CON-group and the proton at the C-2 atom of the monosaccharide unit.

CRGs of different structure types were isolated from the red algae of the family Tichocarpaceae (*Tichocarpus crinitus*) and Gigartinaceae (*Condrus armatus*) collected in the Peter the Great Bay (the Sea of Japan). Morphological and anatomic characteristics of the seaweeds were determined and identified by light microscopy by Prof. E. Titlynov and T. Titlynova (NSCMB FEB RAS). Dried and milled algae (5 g) were suspended in water, and the polysaccharides were extracted at 90 °C for 2 h in a water bath. The crude polysaccharide extracts were filtered through a Vivaflow200 membrane (Sartorius, Gottingen, Germany) with a pore size of 100 kDa (to remove low-molecular-weight impurities), concentrated, dialyzed, and lyophilized. Then, the polysaccharides were separated into gelling KCl- insoluble and nongelling KCl-soluble fractions as described previously, and their structures were established according to the published protocol [33,35].

Elemental analysis of CRG samples was performed using a Vario Micro Cube C, H, S, N analyzer (Elementar, Langenselbold, Germany).

### 3.2. Determination of Molecular Weight

The molecular weight of CH and CRGs was calculated by the Mark–Houwink–Kuhn equation (Equation (1)):

(1) $$\left|ƞ\right|=K_{M}\times M^{\alpha }$$

where |ƞ| is the intrinsic viscosity (calculated by double graphical extrapolation of the lnηsp/C dependence to infinite dilution), M is the molecular weight, and $K_{M}$ and α are empirical constants with values taken from [59,60]. The viscosities of CH solutions in 0.1 M acetate buffer, pH 5.0, containing 0.2 M NaCl, and of CRG solutions in 0.1 M NaCl were determined on a modified Ubbelohde viscometer (SKB Pushchino, Pushchino, Russia) with a capillary diameter of 0.3 mm at 25 °C (measurement accuracy was ±0.1 s).

### 3.3. Film Preparation

Single-layer films were obtained by the application of individual polysaccharide solutions at the same concentrations in the mold placed on a balanced plastic substrate. A solution of 1% CRG was prepared by dissolving CRG in deionized water at 50 °C. A solution of 1% CH was prepared by dissolving CH in a 2% solution of acetic acid.

Composite films were prepared by the layer-by-layer application of a polyion solution. A 1% aqueous solution of CRG was applied in the mold placed on a balanced plastic substrate and dried to a gel-like state, after which a 1% CH solution was applied to the surface of the formed layer. The films were dried at room temperature.

#### 3.3.1. Determination of the PEC Yield

The thus-obtained complex films were a three-layer system containing a polycation (CH), polyanion (CRG), and polyelectrolyte layer formed between layers of two polyions. Water-insoluble PEC was selected from the three-layer film after the dissolution of the unbonded polymers using 2% acetic acid for CH and water for CRG. The PEC yield (%) was calculated as the ratio of the film weight after dissolution of unbound components to the weight of the initial three-layer film.

#### 3.3.2. ECH-Loaded Films

The standardized substance ECH in powder form, registration number in the Russian Federation P N002362/01 (Russian State Register of Drugs, 2016), was obtained in PIBOC FEB RAS. An ethanolic 1% solution of ECH was used as a stock solution. To obtain loaded single-layer films, ECH was added to the polysaccharide solution at a ratio of 1:0.01 (polysaccharide:ECH). Further, the resulting mixture was deposited to the mold placed on a balanced plastic substrate and dried at room temperature without access to light.

Composite ECH-loaded films were prepared by the layer-by-layer application of a polyion solution. ECH was added to 1% CRG solution at a ratio of 1:0.01 (CRG:ECH). Further, the resulting mixture was applied to the mold placed on a balanced plastic substrate and dried to a gel-like state, after which a 1% CH solution was applied to the surface of the formed layer. The films were dried at room temperature for several days without access to light.

### 3.4. Film Characterization

#### 3.4.1. Film Thickness

The thickness of the films was measured at five randomly selected positions, including the middle part, using a digital caliper. The mean ± standard deviation values were calculated.

#### 3.4.2. Moisture Content

An MF-50 Moisture Analyzer (A&D Company Limited, Tokyo, Japan) was used to determine the moisture content of the films. A sample was dried to a constant weight, as defined by a weight change of less than 0.05% per minute at 110 °C.

#### 3.4.3. Mechanical Strength Test of the Films

The system of choice to determine the forces applied on sample specimens was the texture analyzer EZ-LX (Shimadzu, Kyoto, Japan). The system started recording the force and displacement of the probe when in contact with the sample. Films (2 × 2 cm) were fixed by screws between two plates with a cylindrical hole of 12 mm diameter (area of the sample holder hole: Ars = 50.265 mm^2^). The texture analyzer was adjusted to move forward the probe forward with a velocity of 5.0 mm/min. Measurement started when the probe made contact with the sample surface (triggering force). The probe moved on until the film broke apart (at constant speed). The applied force and displacement were registered. All experiments were conducted at room temperature. The mean values of five independent tests ± SD are presented.

Several parameters were calculated (Equations (2)–(4)) to characterize the samples [43].

(2) $$Puncture\;strength=\frac{Force}{Area}$$

where *Force* is the maximum applied force recorded during strain and *Area* is the probe contact area.

(3) $$Elongation\;to\;break=\frac{\sqrt{{\left(a-r\right)}^{2}+b^{2}}+r}{a}$$

where *a* is the radius of the film in the sample holder opening, initial length; *b* is the penetration depth/vertical displacement by the probe; and *r* is the radius of the probe.

(4) $$Energy\;to\;puncture=\frac{AUC}{Volume}$$

where *AUC*, the area under the force-displacement curve, is divided by the total *Volume* of the sample.

### 3.5. Preparation of Mucous Tissue

The inner surface of the pig’s small intestine (immediately after slaughter, it was extracted, washed, and frozen) was used. The mucosal tissue was thawed under mild conditions. After that, the tissue was placed in 0.05 M phosphate buffer, pH 7.0, containing 0.1 M NaCl. The homogeneous morphology without physical defects samples mucosal tissue (about 5 cm^2^) was chosen.

### 3.6. Swelling Test on the Mucosal Surface

Films 1 × 1 cm^2^ in size were weighed, placed on the mucosal surface, and kept in closed Petri dishes for 30 min at room temperature (pH 7). The film was then separated from the mucosa and weighed, after which its degree of swelling (DS) was calculated as follows in Equation (5):

(5) $$DS=\frac{W_{2}-W_{1}}{W_{1}}\times 100\%\;$$

where *W_1_* is the weight of the initial film, and *W_2_* is the weight of the swollen film. The swollen film was then dried at room temperature to a constant weight and weighed again (*W_3_*). Film integrity was assessed after contact with the mucosa and subsequent weight loss or gain calculated. The relative mass change was calculated as $\left(W_{3}/W_{1}\right)\times 100\;\left(\%\right)\;$. The experiment was performed in triplicate. The mean values of three independent tests ± SD are presented.

### 3.7. Structural Studies

X-ray diffraction of the film studies was performed in reflection geometry by a DRON–3M diffractometer using Ni-filtered CuKα radiation (λ = 1.5418 Å, 40 kV, 30 mA).

### 3.8. Scanning Electron Microscopy Study

The morphology of film cross-sections was studied by scanning electron microscopy. The films were cut using scissors, mounted on the surface of aluminum tables, and sprayed with chromium. Samples were analyzed with a Carl Zeiss Sigma 300 VP scanning electron microscope (Carl Zeiss Ltd., Cambridge, UK) at an acceleration voltage of 5.00 kV. Micrographs for sample cross-sections were obtained at 20,000× magnification.

## 4. Conclusions

Polymer films are increasingly investigated as a promising pharmaceutical form for drug delivery due to advantages such as their biocompatibility, form of use, retention time, ease of storage, and stability. In this work, both single-layer films obtained based on CRG and CH and three-layer CRG:PEC:CH were studied. Three-layer films were obtained by a simple and fast method, which excludes the stage of washing from the initial polysaccharides. This method makes it possible to obtain systems independent of the substrate with different surface properties. The mechanical properties analysis showed that their parameters meet the requirements for materials for use as DDS. Κ/β-, λ-, and x-CRGs and CH are capable of forming thin polymer films and among them, κ/β-CRG, which contains a lot of 3,6-anhydrogalactose and a small number of sulphates, forms films with the greatest tensile strength. Multilayer films are stronger than single-layer CRG films due to PEC formation. The value of the resulting puncture strength of the obtained films is similar to those of commercial samples of films and reference soft tissues described in the literature. The mechanical properties of the films are well correlated with their ability to swell on mucous tissue. The films of x- and λ-CRGs, which have the lowest puncture strength and energy, show the greatest swelling, whereas an increase in the mechanical properties of films due to the formation of PEC leads to a decrease in their swelling ability. According to SEM data, three-layer films, in contrast to the films of the initial CRG, are more densely packed structures, which probably explains their higher mechanical properties and a significant decrease in the degree of swelling on the mucous tissue.

The films with a minimal degree of swelling on the mucous membrane (three-layer films and a chitosan film) presumably have a low drug release rate. It can prolong of the drug effect and reduce the frequency of administration. At the same time, the high solubility of the films upon contact with the mucous membrane (λ-CRG film) can be an advantage in the case when it is required to achieve a high local concentration of drugs in the lesion focus.

The inclusion of ECH in polysaccharide films was studied. The addition of ECH to the film composition insignificantly reduced the strength characteristics and increased their swelling capacity on the mucosal surface. All studied films exhibited mucoadhesive properties. These data allow us to consider the obtained films as mucoadhesive carriers of drugs with prolonged release of the dosage form onto the mucous surface.

## Figures and Tables

**Table 1:** CRGs characteristic.

Red Algae	Type of CRG	Structure of the Repeating Disaccharide Unit	MW, kDa	Elemental Composition, %		
				C	H	S
*Chondrus armatus*	λ	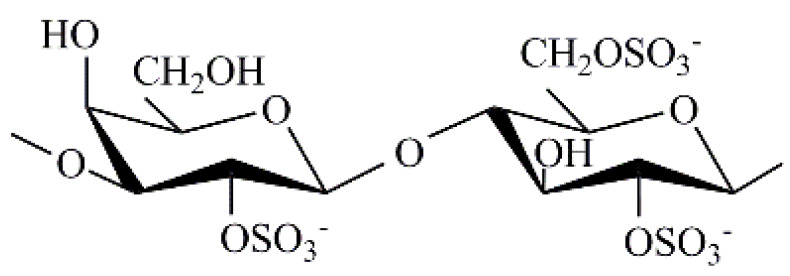	350	23.0	3.8	13.2
*Tichocarpus crinitus*	κ/β	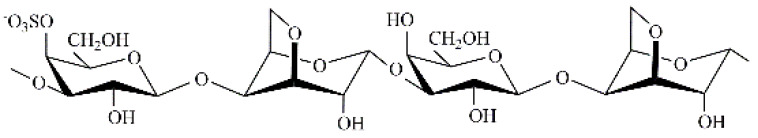	592	28.7	5.0	9.4
	x	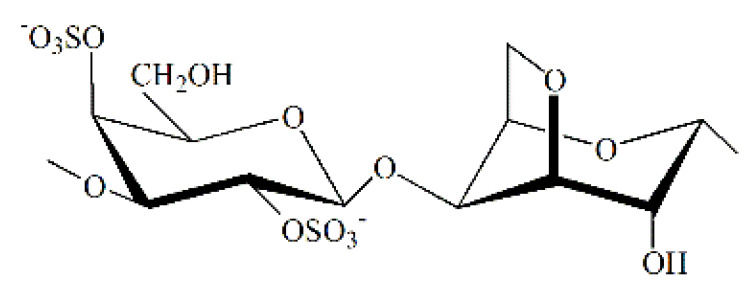	950	25.2	4.1	12.3

**Table 2:** Characteristics of the PEC layer in three-layer films.

PEC	Elemental Composition, %				PEC Content in the Three-Layer Films, %
	C	H	S	N	
κ/β-CRG:CH	33.9	5.17	5.60	2.66	35.2
x-CRG:CH	34.1	5.52	5.93	3.75	29.5
λ-CRG:CH	31.9	5.47	6.85	3.05	22.2

**Table 3:** The relative mass change films after contact with the mucosa.

Sample		$\frac{W_{3}}{W_{1}}\times 100,\;\%$
λ-CRG		90.7 ± 1.3
x-CRG		128.2 ± 1.9
κ/β-CRG		116.8 ± 2.8
CH		108.0 ± 4.3
λ-CRG:PEC:CH	CRG side	94.5 ± 2.0
	CH side	106.2 ± 0.3
x-CRG:PEC:CH PEC	CRG side	102.6 ± 1.7
	CH side	160.3 ± 0.1
κ/β-CRG:PEC:CH	CRG side	144.2 ± 12.2
	CH side	126.0 ± 1.6

**Table 4:** The relative mass change films after contact with the mucosa.

Sample		$\frac{W_{3}}{W_{1}}\times 100,\;\%$
CH(ECH)		176.3 ± 9.9
x-CRG(ECH)		142.7 ± 0.6
κ/β-CRG(ECH)		128.9 ± 3.9
x-CRG(ECH):PEC:CH	CRG side	103.2 ± 5.8
	CH side	135.6 ±1.2
κ/β-CRG (ECH):PEC:CH	CRG side	120.1 ± 2.0
	CH side	124.6 ± 4.6

## Appendix A

**Table A1:** ^1^H-и ^13^C-NMR data polysaccharide from *T. crinitus*.

Type of CRG	Ms Unit		^1^H Chemical Shift (in ppm)				
		H-1	H-2	H-3	H-4	H-5	H-6
κ	G4S	4.68	3.65	4.01	4.87	-	3.83
	DA	5.13	4.01	4.55	4.62	4.66	4.23
β	G	4.61	3.62	3.86	4.128	3.73	3.83
	DA′	5.11	4.08	4.53	4.62	4.66	4.18
			**^13^C Chemical Shift**				
		**C-1**	**C-2**	**C-3**	**C-4**	**C-5**	**C-6**
κ	G4S	103.1	70.0	79.0	74.0	73.86	61.17
	DA	95.1	69.64	80.15	79.10	78.08	69.54
β	G	102.7	70.1	80.82	66.15	75.24	61.17
	DA′	94.4	70.04	79.23	78.57	76.75	69.41
